# Supplementary material for: Interspecies Recombination-Led Speciation of a Novel Geminivirus in Pakistan
Source: Viruses. 2022 Sep 30;14(10):2166. doi: 10.3390/v14102166 (PMC9612148; doi:10.3390/v14102166)
Supplement: Supplementary file 1 [file viruses-14-02166-s001.zip › viruses-1836956-supplementary.pdf]

Supplementary Data

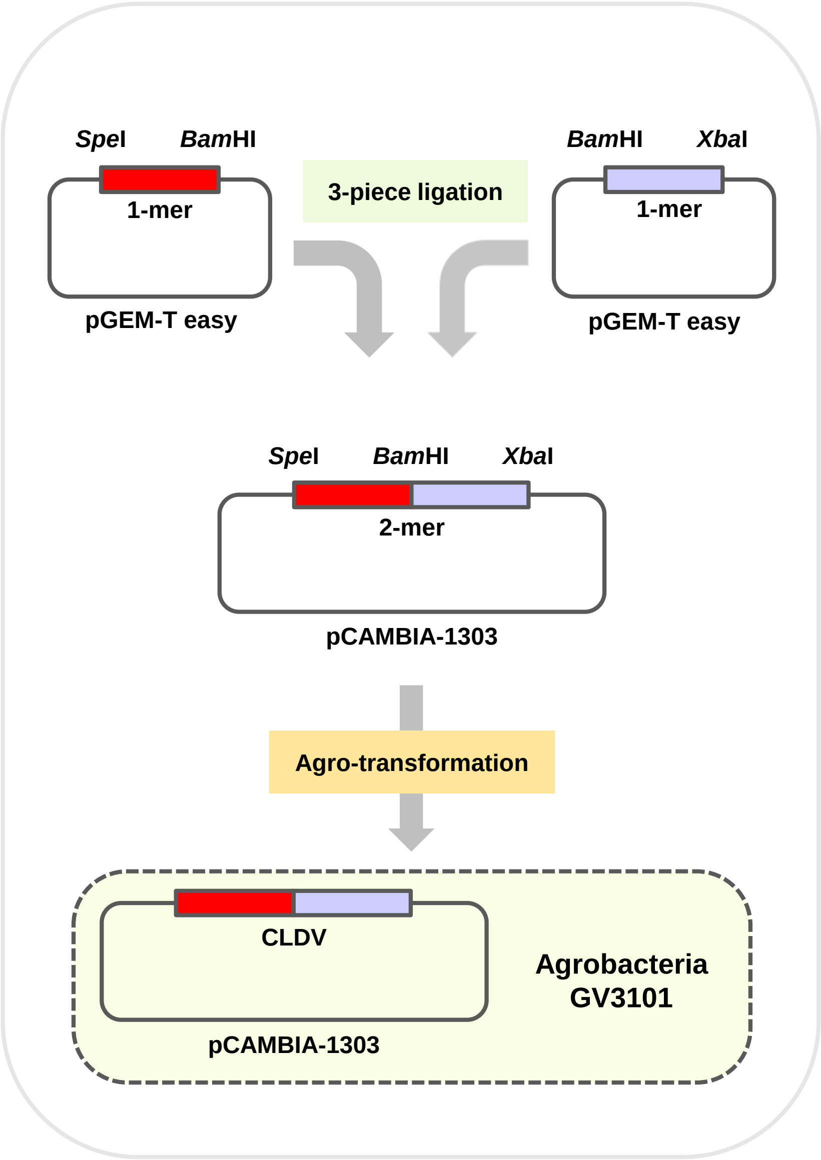

IC1 (*Spe*I - *Bam*HI) 1424
ACTAGT
TTTGGCAATCGGTGTCTCACAAACTTGGCATATCAATTGGTGTCTGGGGTCTTATTTATACCTGGACACCAAATGGCAATTTGGTAATTCAGA
AACTTTAATTTGAATTTTGAAATTCAAAATTCCCAAAAGCGGCCATCCGTATAATATTACCGGATGGCCGCGCTTTTTTGTCCCCTCGTGGGCC
CTACCATGTGCACCATGGACACATGGTCCAATCAAAAGCACTCCTGAAAGCTTGATTGTTTTGTGGGCCCCATATATAATTGCTTGCTGAGTA
AGTCTGTTGTAAACATGTGGGACCCACTTTTGAATGAGTTTCCAGAAACTGTTCATGGGTTTAGGTGTATGCTAGCAATTAAATACTTGCAGC
TAGTAGAAAATACGTATTCCCCAGACACTCTGGGATACGATTTAATTAGGGATTTGATTTCAGTAATAAGGGCTAGGAATTATGTCGAAGCG
ACCAGCAGATATAATCATTTCCACGCCCGCCTCGAAGGTACGCCGCCGTCTCAACTTCGACAGCCCATATGCGAGCCGTGCTGCTGCCCCCAT
TGTCCGCGTCACAAAGGCAAGGGCATGGGCGAACAGGCCCATGAACAGAAAGCCCAGGATGTACAGGATGTACAGAAGCCCAGATGTTCC
GAGGGGATGTGAAGGCCCATGCAAGGTCCAGTCATTTGAGTCCAGACATGATATCCAGCACATTGGTAAAGTCATGTGTGTTAGTGATGTTA
CTCGTGGTATTGGGCTGACCCACAGGGTTGGCAAGAGGTTCTGTGTTAAGTCCGTTTATGTTCTGGGCAAGATCTGGATGGATGAGAACATC
AAGACTAAGAATCATACGAATAGTGTTATGTTTTTTCTTGTTAGGGATCGTAGGCCTGTTGACAAGCCTCAAGATTTTGGTGAGGTGTTTAAC
ATGTTTGATAATGAGCCCAGCACGGCGACTGTGAAGAATGTTCATCGTGATAGGTACCAGGTATTAAGGAAGTGGCACGCAACTGTGACAG
GTGGCCTGTATGCATCGAAGGAGCAGGCTCTCGTGAAGAAGTTTATTAGGGTTAATAATTATGTTGTGTACAACCAGCAAGAGGCTGGCAAG
TATGAGAATCATACTGAGAATGCATTGATGTTGTATATGGCGTGTACCCACGCCTCTAACCCTGTGTATGCCACACTGAAGATACGGATCTAT
TTTTATGATTCAGTATCGAATTAATAAAATTTAAATTTTATATCATGATCCTCAATTACATCAATTGTGCCCTCAAGTACATCATATAATACATG
TTTAAATGCCCTAATACAATTATTTATACTAATCACTCCTAATCTATCTAAATATCTTAAAACATGAGTCTTAAAGACTCTTAAGAAATGCCCAG
TCTGAGGATGTAAACGAGTGT**GGATCC**

IC2 (*Bam*HI - *Xba*I) 1650
GGATCCTCAAGCCCAAGAAACACTTCATTATCCCCAGCTCCTTCCTGAGGTTGTGATTGAACTGGACCCTGATGTGGATGATGTCGTGGTTCA
TGTTGAGTGGCCTTTTGTCGTGGTTGAGGATCTTGAAATACAGGGGATTTGGGACTTCCCAGATATACACGCCATTCATTGCCTGAGCTGCAG
TGATGGATTCCCCTGTGCGTGAATCCATGGTTGTGGCAGTTGATGTGTACGTAGTATGAGCAGCCACACTCGAGGTCAACCCTCTTACGCCGG
ATGGCTCTACGCTTGGCTAGCCTGTGTTGGACCTTGATGGGCACCTGAGTACAGTGGCTCTGTGAGGGTGATGAATGCTGCATTGTGTATAG
CCCAAGACTTCAGTGCTGAGTTCTTTTCCTCATCGAGGAACTCTTTATAGCTGGAATTGGGCCCAGGATTGCATAGGAAGATTGTGGGAATGC
CCCCTTTAATTTGAACTGGCTTCCCGTACTTGGTGTTACTTTGCCAGTCCCTCTGGGCCCCCATGAATTCTTTAAAGTGCTTTAGGTAGTGGGG
GTCTACGTCATCAATGACGTTGTACCATGCATCATTTGAATAGATCTTAGGGCTCAGATCTAAATGGCCACATAGGTAATTATGTGGACCCAG
TGACCTAGCCCACATTGTCTTCCCCGTACGACTATCACCCTCAATGACGATACTTTTAGGTCTCAATGGCCGCGCAGCGGGACCCATCACATTT
TCAGAGGCCCATTCCTCTATGGCCTCTGGAACTTGATCGAAGGAAGAAGAAAGAAAAGGGGAAACATAAACCTCCATTGGAGGTGCAAAAA
TCCTATCTAAATTAGCATTTAAATTATGATATTGAAAAATATATTTTTCTGGGAGTTTTTCCCTTATTATAGCCATTGCAGCTTCTTTAGAACCTG
CATTTAGTGCTTCGGCAGCAGCATCATTAGCTGTCTGTTGACCTCCTCGTGCAGATCTTCCATCGATCTGGAATTCTCCCCAGTCAAGGGTGTC
TCCGTCCTTGTCGATGTAGGACTTGACGTCGGTGCTGGATTTAGCTCCCTGAATGTTTGGATGGAAGTGTGCTGACCTGTTTGGGGAGACCAG
GTCGAAGAATCGTTGATTCTTGCACTGATATTTCCCTTCGAACTGGATGAGCACATGGAGATGAGGGCTCCCATCTTCGTGTAGTTCTCTGCA
GATCTTGATGTATTTTTTGTTTGTTGGGGTTTCGAGTGCTTCAAGTTGGGAAAGTGCTTCCTCTTTAGTAAGGGAGCATTTAGGATAAGTAAG
GAAATAATTTTTGGCGTTAATTCTGAAGGAATTAGCACGTGGCATTTTGGCAATCGGTGTCTCACAAACTTGGCATATCAATTGGTGTCTGGG
GTCTTATTTATACCTGGACACCAAATGGCAATTTGGTAATTCAGAAACTTTAATTTGAATTTTGAAATTCAAAATTCCCAAAAGCGGCCATCCG
TATAATATTACCGGATGGCCGCGCTTTTTTGTCCCCTCGTGGGCCCTACCATGTGCACCATGGACACATGGTCCAATCAAAAGCACTCCTGAA
AGCTTGATTGTTTTGTGGGCCCCATATATAATTGCTTGCTGAGTAAGTCTGTTGTAAAC**TCTAGA**

Supplementary Figure S1. Schematic diagram of IC construction of CLDV. 1.1 mer IC was constructed by the addition of restriction enzyme sites i.e., *Spe*I at the start of IC1 and *Xba*I at the end of IC2. *Bam*HI is the common point of digestion (end of IC1; start of IC2) existing naturally in the sequence. The sequences of both IC1 and IC2 have been shown as well in the box on the right side. Restriction enzymes in the sequences (IC1 and IC2) are shown in bold letters. Both IC1 and IC2 are ligated with digested pCambia-1303 followed by the transformation into Agrobacterium strain GV3101.

```
AC1
1  130
AC1_AEV    MAAPNRFKIN AKNYFLTYPK CSLTKEEALS QLLNLQTPTN KKYIKICREL HEDGSPHLHV LIQFEGKYQC KNQRFFDLVS PNRSAHFHPN IQGAKSSTDV KSYIDKDGDT LEWGEFQIDG RSARGGQQTA
AC1_CLDV   MPRANSFRIN AKNYFLTYPK CSLTKEEALS QLEALETPTN KKYIKICREL HEDGSPHLHV LIQFEGKYQC KNQRFFDLVS PNRSAHFHPN IQGAKSSTDV KSYIDKDGDT LDWGEFQIDG RSARGGQQTA
Consensus  MaaaNrFrIN AKNYFLTYPK CSLTKEEALS QLeaL#TPTN KKYIKICREL HEDGSPHLHV LIQFEGKYQC KNQRFFDLVS PNRSAHFHPN IQGAKSSTDV KSYIDKDGDT L#WGEFQIDG RSARGGQQTA

131  260
AC1_AEV    NDAAAEALNA GSKEAAMAII KEKLPEKFIF QYHNLNANLD RIFSPPLEVY VSPFLSSSFD QVPEELEEWV SENVMAAAAR PLRPQSIVIE GDSRTGKTMW ARSLGPHNYL CGHLDLSPKV YSNDAWYNVI
AC1_CLDV   NDAAAEALNA GSKEAAMAII REKLPEKYIF QYHNLNANLD RIFAPPMEVY VSPFLSSSFD QVPEAIEEWA SENVMGPAAR PLRPKSIVIE GDSRTGKTMW ARSLGPHNYL CGHLDLSPKI YSNDAWYNVI
Consensus  NDAAAEALNA GSKEAAMAII rEKLPEK%IF QYHNLNANLD RIFaPP$EVY VSPFLSSSFD QVPEaiEEWa SENVMaaAAR PLRPqSIVIE GDSRTGKTMW ARSLGPHNYL CGHLDLSPK! YSNDAWYNVI

261  361
AC1_AEV    DDVDPHYLKH FKEFMGAQRD WQSNTKYGKP VQIKGGIPTI FLCNPGPNSS YKEFLDEEKN NALKNWALKN AIFVTLEGPL YSGSNQSAAQ ASQEGDEAST C
AC1_CLDV   DDVDPHYLKH FKEFMGAQRD WQSNTKYGKP VQIKGGIPTI FLCNPGPNSS YKEFLDEEKN SALKSWAIHN AAFITLTEPL YSGAHQGPTQ ASQA
Consensus  DDVDPHYLKH FKEFMGAQRD WQSNTKYGKP VQIKGGIPTI FLCNPGPNSS YKEFLDEEKN nALKnWAihN AaF!TLeePL YSGanQgaaQ ASQa...... .

AC2
1  130
AC2_RLCV   MQHSSPSQSH CTQVPIKVQH RLAKRRAIRR KRVDLECGCS YYVHINCHNH GFTHRGIHHC SSGNEWRVYL GSPKSPVFQD PQPRPKATPH EPRHHPHQGP VQSQPQEGAG DNEVFLGLED PHSFTSSDWA
AC2_CLDV   MQHSSPSQSH CTQVPIKVQH RLAKRRAIRR KRVDLECGCS YYVHINCHNH GFTHRGIHHC SSGNEWRVYL GSPKSPVFQD PQPRQKATQH EPRHHPHQGP VQSQPQEGAG DNEVFLGLED PHSFTSSDWA
Consensus  MQHSSPSQSH CTQVPIKVQH RLAKRRAIRR KRVDLECGCS YYVHINCHNH GFTHRGIHHC SSGNEWRVYL GSPKSPVFQD PQPRqKATqH EPRHHPHQGP VQSQPQEGAG DNEVFLGLED PHSFTSSDWA
131
AC2_RLCV   FLKSL
AC2_CLDV   FLKSL
Consensus  FLKSL

AC3
1  130
AC3_RLCV   MDSRTGESIT AAQAMNGVYI WEVPNPLYFK ILNHDQRPLL MNHDIIHIRV QFNHNLRKEL GIMKCFLGLR IHTRLHPQTG HFLRVFKTHV LRYLDRLGVI SINNCIRAFK HVLYDVLEGT IDVIEDHDIK
AC3_CLDV   MDSRTGESIT AAQAMNGVYI WEVPNPLYFK ILNHDKRPLN MNHDIIHIRV QFNHNLRKEL GIMKCFLGLR IHTRLHPQTG HFLRVFKTHV LRYLDRLGVI SINNCIRAFK HVLYDVLEGT IDVIEDHDIK
Consensus  MDSRTGESIT AAQAMNGVYI WEVPNPLYFK ILNHDqRPLn MNHDIIHIRV QFNHNLRKEL GIMKCFLGLR IHTRLHPQTG HFLRVFKTHV LRYLDRLGVI SINNCIRAFK HVLYDVLEGT IDVIEDHDIK
131
AC3_RLCV   FKFY
AC3_CLDV   FKFY
Consensus  FKFY

AC4
1  85
AC4_AEV    MGALISMCSS SSKGNISARI NDSSTWYPQT GQHISIRTFR ELNPAPTSSP TSTRTETPSN GENSRSMEDL HEEVNRQLMM LLQKP
AC4_CLDV   MGALISMCSS SSKGNISARI NDSSTWSPQT GQHTSIQTFR ELNPAPTSSP TSTRTETPLT GENSRSMEDL HEEVNRQLMM LLPKH
Consensus  MGALISMCSS SSKGNISARI NDSSTWsPQT GQHiSIrTFR ELNPAPTSSP TSTRTETPln GENSRSMEDL HEEVNRQLMM LLqKh

AV1
1  130
CP_CYMV    MSKRPADIII STPASKVRRR LNFDSPYASR AAAPIVRVTK ARAWANRPMN RKPRMYRMYR SPDVPRGCEG PCKVQSFESR HDIQHIGKVM CVSDVTRGIG LTHRVGKRFC VKSVYVLGKI WMDENIKTKN
CP_CLDV    MSKRPADIII STPASKVRRR LNFDSPYASR AAAPIVRVTK ARAWANRPMN RKPRMYRMYR SPDVPRGCEG PCKVQSFESR HDIQHIGKVM CVSDVTRGIG LTHRVGKRFC VKSVYVLGKI WMDENIKTKN
Consensus  MSKRPADIII STPASKVRRR LNFDSPYASR AAAPIVRVTK ARAWANRPMN RKPRMYRMYR SPDVPRGCEG PCKVQSFESR HDIQHIGKVM CVSDVTRGIG LTHRVGKRFC VKSVYVLGKI WMDENIKTKN

131  256
CP_CYMV    HTNSVMFFLV RDRRPVDKPQ DFGEVFNMFD NEPSTATVKN VHRDRYQVLR KWHATVTGGL YASKEQALVK KFIRVNNYVV YNQQEAGKYE NHTENALMLY MACTHASNPV YATLKIRIYF YDSVSN
CP_CLDV    HTNSVMFFLV RDRRPVDKPQ DFGEVFNMFD NEPSTATVKN VHRDRYQVLR KWHATVTGGL YASKEQALVK KFIRVNNYVV YNQQEAGKYE NHTENALMLY MACTHASNPV YATLKIRIYF YDSVSN
Consensus  HTNSVMFFLV RDRRPVDKPQ DFGEVFNMFD NEPSTATVKN VHRDRYQVLR KWHATVTGGL YASKEQALVK KFIRVNNYVV YNQQEAGKYE NHTENALMLY MACTHASNPV YATLKIRIYF YDSVSN

AV2
1  121
AV2_RLCV   MWDPLLNEFP ETVHGFRCML AIKYLQLVEN TYSPDTLGYD LIRDLISVIR ARNYVEATSR YNHFHARLEG TPPSQLRQPI CEPCCCPHCP RHKGKGMGEQ AHEQKAQDVQ DVQKPRCSEG M
AV2_CLDV   MWDPLLNEFP ETVHGFRCML AIKYLQLVEN TYSPDTLGYD LIRDLISVIR ARNYVEATSR YNHFHARLEG TPPSQLRQPI CEPCCCPHCP RHKGKGMGEQ AHEQKAQDVQ DVQKPRCSEG M
Consensus  MWDPLLNEFP ETVHGFRCML AIKYLQLVEN TYSPDTLGYD LIRDLISVIR ARNYVEATSR YNHFHARLEG TPPSQLRQPI CEPCCCPHCP RHKGKGMGEQ AHEQKAQDVQ DVQKPRCSEG M
```

Supplementary Figure S2. Alignment of Each ORF of CLDV with their respective identical ORF. AEV was aligned with CLDV in the cases of AC1 and AC4. Alignment of RLCV with CLDV was shown in the cases of AC2, AC3, and AV2. In the case of AV1, CYMV was aligned with CLDV.

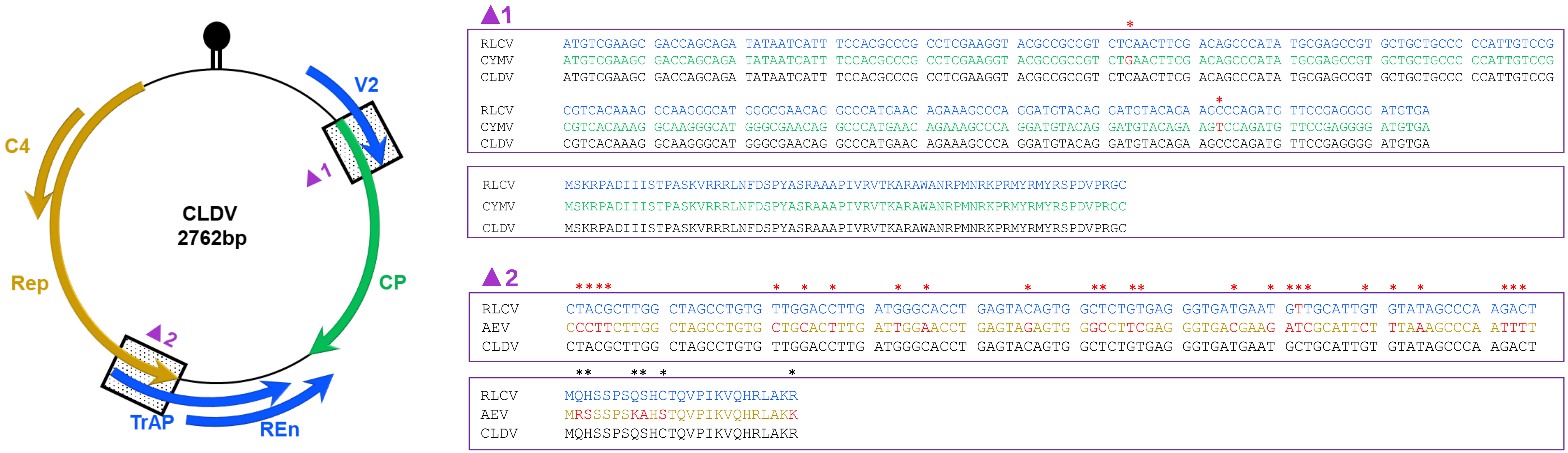

Supplementary Figure S3. Analysis of the common region between the ORFs: (Δ1) coat protein-movement protein and (Δ2) replication protein-transcriptional activator protein. The common region of CLDV ORFs was compared with their respective contendents i.e., RLCV and CYMV in the case of Δ1; RLCV and AEV in the case of Δ2. Analysis was done on both i.e., nucleotide and amino acid levels. In the case of Δ1, RLCV showed more identity with CLDV than CYMV. In the case of Δ2, RLCV showed more identity to CLDV than AEV. The asterisk in red color (*) shows the variations in the nucleotides whereas the asterisk in black color (*) shows the variations in the comparison at the amino acid level.

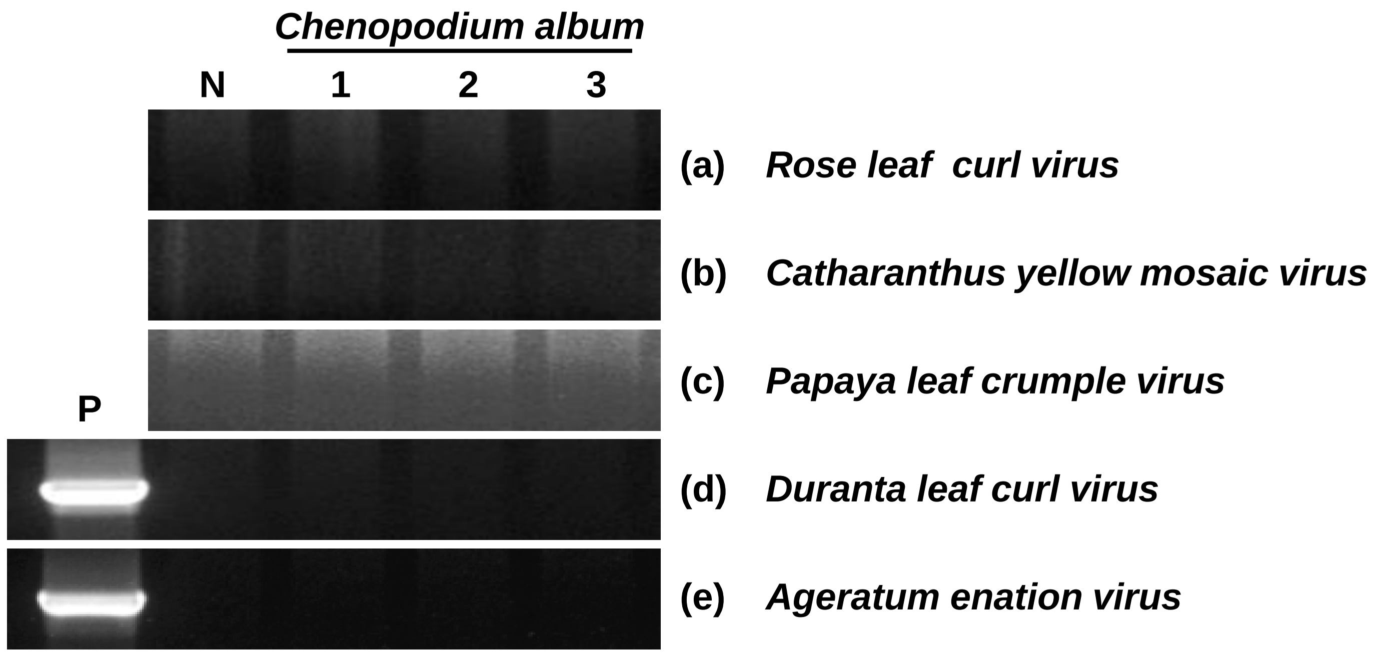

Supplementary Figure S4. The exploration of the intriguing genomic composition of the newly detected virus (CLDV). The primers were constructed based on one of the ORF sequences of the recombinant viruses rather than their identical ORFs in the new virus genomic composition. (a) RLCV, (b) CYMV, (c) PaLCrV, (d) DLCV, and (e) AEV couldn't be detected in the infected samples using the specific ORF-based primers. P: positive control (only for DLCV and AEV as didn't have positive control for RLCV, CYMV, PaLCrV), N: Negative control, Lanes 1-3: infected samples (*C. album*).

Supplementary Table S1. Sequences downloaded from NCBI GenBank and used in this study.

#	Virus	Total sequences	Accession numbers (NCBI GenBank)
1	*Ageratum enation virus* (AEV)	30	LT716985, LT716984, KP725057, KM262823, JF728867, KC818421, JX436473, HE861940, JQ911765, FN794201, FN543099, AM701770, MN187418, NC_003434, MG686551, KY089033, KM383735, KC589699, AM698011, AM261836, AJ437618, GQ268327, KM262822, KM066975, JQ911767, KJ488991, KJ488990, JF728860, JX436472, FN794198
2	*Catharanthus yellow mosaic virus* (CYMV)	06	LK028573, MH643737, LK028570, HE580234, HE580235, LN864815
3	*Duranta leaf curl virus* (DLCV)	05	MN537564, NC_038980, KT948069, MH807202, MN166094
4	*Papaya leaf crumple virus* (PaLCrV)	10	KR052159, MH807203, MH807200, MH807201, KJ028210, KM359408, NC_014707, HM140369, HM140368, HM140367
5	*Rose leaf curl virus* (RLCV)	05	NC_024687, KJ739692, MN746285, GQ478342, KF584008
6	*Chenopodium leaf distortion virus* (CLDV)	01	MN423112

Supplementary Table S2. The sequence homology of each ORFs of CLDV with the genes of its composition.

Virus	*Rep*	*C4*	*TrAP*	*REn*	*CP*	*MP*
Ageratum enation virus	***91***	**94**	61	63	97	91
Rose leaf curl virus	*88*	67	**99**	**99**	96	**100**
Catharanthus yellow mosaic virus	*77*	58	85	87	**100**	99

Supplementary Table S3. Recombination analysis of recombinant CLDV identified in this study.

Recombinant [GenBank]	Break points (5´-3´ nucleotides)	Major parent [GenBank]	Minor parent [GenBank]	Detection	Score	P value
CLDV [MN423112]*	2280-1059	CYMV [MH643737]	RLCV [GQ478342]	RGBMCS3	0.47	2.98×10^{-15}
RLCV [QG478342]	854-1381	DLCV [NC_038980]	CYMV [MH643737]	RGMCS3	0.60	5.78×10^{-20}
CYMV [MH643737]	1861-2686	Unknown	AEV [KC795968]	RGMCS3	0.44	5.89×10^{-13}

*Present study isolate: AEV; Ageratum enation virus, CLDV; Chenopodium leaf distortion virus, CYMV; Catharanthus yellow mosaic virus, RLCV; Rose leaf curl virus.

Supplementary Table S4. Estimation of molecular diversity among PaLCrV, AEV, CYMV, DLCV and RLCV populations.

	Haplotype-nucleotide diversity							Neutrality tests	
Virus	*n*	*S*	*Eta*	*H*	H_d	π	θ_w	Tajima's D	Fi-Lu's F
PaLCrV	10	633	691	08	0.956	0.0651	0.0819	-1.4063	-1.5289
AEV	30	662	768	29	0.998	0.0494	0.0635	-1.1259	-1.3594
CYMV	06	216	223	04	0.867	0.0424	0.0344	1.1617	1.2396
DLCV	05	276	282	04	0.901	0.0442	0.0480	-0.8438	-0.8903
RLCV	05	144	145	03	0.701	0.0257	0.0255	-0.0109	-0.0024

>MN423112_CLDV Full-length sequence

ACCGGATGGCCGCGCTTTTTTGTCCCCTCGTGGGCCCTACCATGTGCACCATGGACACATGGTCCAATCAAAAGCACTCCTGAAAGCTTGATTGT
TTTGTGGGCCCCATATATAATTGCTTGCTGAGTAAGTCTGTTGTAAACATGTGGGACCCACTTTTGAATGAGTTTCCAGAAACTGTTCATGGGTT
TAGGTGTATGCTAGCAATTAAATACTTGCAGCTAGTAGAAAATACGTATTCCCCAGACACTCTGGGATACGATTTAATTAGGGATTTGATTTCAG
TAATAAGGGCTAGGAATTATGTCGAAGCGACCAGCAGATATAATCATTTCCACGCCCGCCTCGAAGGTACGCCGCCGTCTCAACTTCGACAGCCC
ATATGCGAGCCGTGCTGCTGCCCCCATTGTCCGCGTCACAAAGGCAAGGGCATGGGCGAACAGGCCCATGAACAGAAAGCCCAGGATGTACAGGA
TGTACAGAAGCCCAGATGTTCCGAGGGGATGTGAAGGCCCATGCAAGGTCCAGTCATTTGAGTCCAGACATGATATCCAGCACATTGGTAAAGTC
ATGTGTGTTAGTGATGTTACTCGTGGTATTGGGCTGACCCACAGGGTTGGCAAGAGGTTCTGTGTTAAGTCCGTTTATGTTCTGGGCAAGATCTG
GATGGATGAGAACATCAAGACTAAGAATCATACGAATAGTGTTATGTTTTTTCTTGTTAGGGATCGTAGGCCTGTTGACAAGCCTCAAGATTTTG
GTGAGGTGTTTAACATGTTTGATAATGAGCCCAGCACGGCGACTGTGAAGAATGTTCATCGTGATAGGTACCAGGTATTAAGGAAGTGGCACGCA
ACTGTGACAGGTGGCCTGTATGCATCGAAGGAGCAGGCTCTCGTGAAGAAGTTTATTAGGGTTAATAATTATGTTGTGTACAACCAGCAAGAGGC
TGGCAAGTATGAGAATCATACTGAGAATGCATTGATGTTGTATATGGCGTGTACCCACGCCTCTAACCCTGTGTATGCCACACTGAAGATACGGA
TCTATTTTTATGATTCAGTATCGAATTAATAAAATTTAAATTTTATATCATGATCCTCAATTACATCAATTGTGCCCTCAAGTACATCATATAAT
ACATGTTTAAATGCCCTAATACAATTATTTATACTAATCACTCCTAATCTATCTAAATATCTTAAAACATGAGTCTTAAAGACTCTTAAGAAATG
CCCAGTCTGAGGATGTAAACGAGTGTGGATCCTCAAGCCCAAGAAACACTTCATTATCCCCAGCTCCTTCCTGAGGTTGTGATTGAACTGGACCC
TGATGTGGATGATGTCGTGGTTCATGTTGAGTGGCCTTTTGTCGTGGTTGAGGATCTTGAAATACAGGGGATTTGGGACTTCCCAGATATACACG
CCATTCATTGCCTGAGCTGCAGTGATGGATTCCCCTGTGCGTGAATCCATGGTTGTGGCAGTTGATGTGTACGTAGTATGAGCAGCCACACTCGA
GGTCAACCCTCTTACGCCGGATGGCTCTACGCTTGGCTAGCCTGTGTTGGACCTTGATGGGCACCTGAGTACAGTGGCTCTGTGAGGGTGATGAA
TGCTGCATTGTGTATAGCCCAAGACTTCAGTGCTGAGTTCTTTTCCTCATCGAGGAACTCTTTATAGCTGGAATTGGGCCCAGGATTGCATAGGA
AGATTGTGGGAATGCCCCCTTTAATTTGAACTGGCTTCCCGTACTTGGTGTTACTTTGCCAGTCCCTCTGGGCCCCCATGAATTCTTTAAAGTGC
TTTAGGTAGTGGGGGTCTACGTCATCAATGACGTTGTACCATGCATCATTTGAATAGATCTTAGGGCTCAGATCTAAATGGCCACATAGGTAATT
ATGTGGACCCAGTGACCTAGCCCACATTGTCTTCCCCGTACGACTATCACCCTCAATGACGATACTTTTAGGTCTCAATGGCCGCGCAGCGGGAC
CCATCACATTTTCAGAGGCCCATTCCTCTATGGCCTCTGGAACTTGATCGAAGGAAGAAGAAAGAAAAGGGGAAACATAAACCTCCATTGGAGGT
GCAAAAATCCTATCTAAATTAGCATTTAAATTATGATATTGAAAAATATATTTTTCTGGGAGTTTTTCCCTTATTATAGCCATTGCAGCTTCTTT
AGAACCTGCATTTAGTGCTTCGGCAGCAGCATCATTAGCTGTCTGTTGACCTCCTCGTGCAGATCTTCCATCGATCTGGAATTCTCCCCAGTCAA
GGGTGTCTCCGTCCTTGTCGATGTAGGACTTGACGTCGGTGCTGGATTTAGCTCCCTGAATGTTTGGATGGAAGTGTGCTGACCTGTTTGGGGAG
ACCAGGTCGAAGAATCGTTGATTCTTGCACTGATATTTCCCTTCGAACTGGATGAGCACATGGAGATGAGGGCTCCCATCTTCGTGTAGTTCTCT
GCAGATCTTGATGTATTTTTTGTTTGTTGGGGTTTCGAGTGCTTCAAGTTGGGAAAGTGCTTCCTCTTTAGTAAGGGAGCATTTAGGATAAGTAA
GGAAATAATTTTTGGCGTTAATTCTGAAGGAATTAGCACGTGGCATTTTGGCAATCGGTGTCTCACAAACTTGGCATATCAATTGGTGTCTGGGG
TCTTATTTATACCTGGACACCAAATGGCAATTTGGTAATTCAGAAACTTTAATTTGAATTTTGAAATTCAAAATTCCCAAAAGCGGCCATCCGTA
TAATATT
